# LILRA2 Selectively Modulates LPS-Mediated Cytokine Production and Inhibits Phagocytosis by Monocytes

**DOI:** 10.1371/journal.pone.0033478

**Published:** 2012-03-30

**Authors:** Hao K. Lu, Ainslie Mitchell, Yasumi Endoh, Taline Hampartzoumian, Owen Huynh, Luis Borges, Carolyn Geczy, Katherine Bryant, Nicodemus Tedla

**Affiliations:** 1 Inflammation and Infection Research Centre, School of Medical Sciences, Department of Pathology, University of New South Wales, United Kingdom; 2 South Western Sydney Clinical School, Liverpool Hospital, Sydney, Australia; 3 Amgen Seattle, Seattle, Washington, United States of America; National Institute of Environmental Health Sciences, United States of America

## Abstract

The activating immunoglobulin-like receptor, subfamily A, member 2 (LILRA2) is primarily expressed on the surface of cells of the innate immunity including monocytes, macrophages, neutrophils, basophils and eosinophils but not on lymphocytes and NK cells. LILRA2 cross-linking on monocytes induces pro-inflammatory cytokines while inhibiting dendritic cell differentiation and antigen presentation. A similar activating receptor, LILRA4, has been shown to modulate functions of TLR7/9 in dendritic cells. These suggest a selective immune regulatory role for LILRAs during innate immune responses. However, whether LILRA2 has functions distinct from other receptors of the innate immunity including Toll-like receptor (TLR) 4 and FcγRI remains unknown. Moreover, the effects of LILRA2 on TLR4 and FcγRI-mediated monocyte functions are not elucidated. Here, we show activation of monocytes via LILRA2 cross-linking selectively increased GM-CSF production but failed to induce IL-12 and MCP-1 production that were strongly up-regulated by LPS, suggesting functions distinct from TLR4. Interestingly, LILRA2 cross-linking on monocytes induced similar amounts of IL-6, IL-8, G-CSF and MIP-1α but lower levels of TNFα, IL-1β, IL-10 and IFNγ compared to those stimulated with LPS. Furthermore, cross-linking of LILRA2 on monocytes significantly decreased phagocytosis of IgG-coated micro-beads and serum opsonized *Escherichia coli* but had limited effect on phagocytosis of non-opsonized bacteria. Simultaneous co-stimulation of monocytes through LILRA2 and LPS or sequential activation of monocytes through LILRA2 followed by LPS led lower levels of TNFα, IL-1β and IL-12 production compared to LPS alone, but had additive effect on levels of IL-10 and IFNγ but not on IL-6. Interestingly, LILRA2 cross-linking on monocytes caused significant inhibition of TLR4 mRNA and protein, suggesting LILRA2-mediated suppression of LPS responses might be partly via regulation of this receptor. Taken together, we provide evidence that LILRA2-mediated activation of monocytes is significantly different to LPS and that LILRA2 selectively modulates LPS-mediated monocyte activation and FcγRI-dependent phagocytosis.

## Introduction

The leukocyte immunoglobulin-like receptor A2 (LILRA2) belongs to a highly homologous family of activating and inhibitory receptors constitutively expressed on the surface of leukocytes [1], [2]. LILRA2 is an activating receptor which is preferentially expressed on monocytes, macrophages, neutrophils, eosinophils and basophils [3]–[5], but not on lymphocytes or NK cells, suggesting that this receptor may play a role in the innate immune responses. LILRA2 contains a short cytoplasmic tail and a highly charged transmembrane domain that associates with the common gamma chain of the Fc receptor (FcγR) and transduces activation signals via an immunoreceptor tyrosine-based activating motif (ITAM) [3]. Cross-linking of LILRA2 on the surface of leukocytes recruits protein tyrosine kinase signaling molecules that lead to cellular activation [3]. On monocytes, engagement of LILRA2 causes calcium influx and increased production of pro-inflammatory cytokines [3], [6], [7]. Similarly, cross-linking of LILRA2 on the surface of eosinophils and basophils causes significant degranulation of these cells and increased production of pro-inflammatory mediators and immune regulatory cytokines suggesting that it may play a role in modulation of inflammatory responses [4], [5]. This is supported by findings of extensive expression of LILRA2 protein on the surface of macrophages in synovial tissue of patients with active rheumatoid arthritis [8] and on the skin of patients with leprosy [9]. Interestingly, the level of LILRA2 expression is significantly higher in patients with lepromatous leprosy, a disease characterized by Th2 biased immune response leading to severe inflammation but poor bacterial killing as compared to tuberculoid leprosy, which is characterized by Th1 biased response with minimal inflammation but efficient bacterial killing [9]. This suggests that LILRA2 might be an immune modulator that may favor Th2 immune response rather than acting as a simple activating receptor. Moreover, LILRA2 has been shown to inhibit dendritic cell differentiation and reduce antigen presentation to T cells *in vitro* [6] suggesting a more complex functions.

Toll-like receptor-4 (TLR4) is expressed on monocytes and macrophages and plays a key role in innate immune responses by recognizing bacterial lipopolysaccharide (LPS) [10], [11]. The high affinity IgG receptor 1, FcγR1 (aka CD64) is also abundantly expressed on monocytes and is important in innate immune responses against pathogens [12]. Aggregation of this receptor on the surface of moncytes by antibodies and multivalent antigens induces phagocytosis of IgG-coated antigens, causes granule secretion and induces production of reactive oxygen species [13]–[16].

This study is aimed at elucidating whether LILRA2, which is constitutively co-expressed with TLR4 and FcγR1 on the surface of monocytes, has functions different to these receptors and/or modulates their functions. We show that cross-linking of LILRA2 induces pro-inflammatory and immune regulatory cytokines by monocytes that are different to cytokines induced through the engagement of TLR4. We also show that LILRA2 strongly and selectively modulates LPS induced activation of monocytes, partly via regulation of TLR4 expression and inhibits IgG-dependent particle phagocytosis. These results suggest that LILRA2 is not only an important activating receptor in monocytes and macrophages but also a key immune regulatory molecule capable of fine tuning innate immune responses.

## Materials and Methods

### Primary antibodies and reagents

Specific mouse IgG_1_ monoclonal antibody (mAb) against LILRA2 was generated in BALB/c mice by immunization with LILR-Fc fusion protein containing the extracellular domains fused to the Fc region of human IgG_1_ by L Borges at Amgen Inc (Seattle, WA). This antibody was carefully selected for its specific reaction to LILRA2 but not to the Fc portion of the fusion protein or other LILR-Fc fusion proteins [17], [18]. Mouse anti-CD14-PE and FITC, CD16-Percp, CD3-PE, CD56-Percp, CD4-Percp, CD8-APC and CD19-APC mAbs against peripheral blood leukocyte surface markers and corresponding isotype and flurochrome-matched IgG controls were purchased from Pharmingen (Mountain View, CA). Mouse anti-CD9-PE and mouse anti-FcεRI-PE were purchased from Beckman Coulter Australia (Mount Waverly, Vic) and Serotec (Abacus ALS Australia) respectively. FITC-conjugated anti-TLR4 mAb was purchased from e-Biosciences (San Diego, CA). Unconjugated irrelevant mouse IgG_1_-negative control and mouse IgG_1_ anti-MHC class I mAbs were purchased from Sigma (Australia) and Pharmingen respectively. Zenon mouse IgG_1_ labeling kit was used to directly conjugate anti-LILRA2 with Alexa 488 (Molecular Probes Inc, Eugene, OR). Levels of endotoxin in anti-LILRA2 cross-linking mAb, corresponding IgG_1_ and anti-MHC-class I controls and media were tested using Limulus Amebocyte Lysate (LAL) Gel Clot method (Associates of Cape Cod Inc. Falmouth, MA, USA). All reagents showed endotoxin levels less than the sensitivity of the test (0.03 EU/ml).

### Isolation of peripheral blood mononuclear cells (PBMC) and monocytes

Buffy coats (100 ml) from peripheral venous blood, a by-product of packed red cell preparation for transfusion was obtained from healthy blood donors through the Australian Blood Services (Australian Red Cross, Sydney). Alternatively 50 ml peripheral venous blood was collected from 20–40 year old healthy volunteers. The buffy coat or peripheral blood was mixed with acid citrate dextrose and used to isolate PBMCs and polymorphonuclear cells (PMN) using dextran sedimentation and density gradient centrifugation (Ficoll-Paque Plus, Amersham Biosciences, Uppsala, Sweden) [19]. Monocytes were then negatively selected from the freshly isolated PBMCs using magnetic beads (Monocyte isolation kit, Miltenyi Biotec) with 95±3.2% purity as confirmed by flow cytometry using anti-CD14 mAb. High purity eosinophils and basophils were negatively selected from fresh PMN and PBMC respectively using magnetic beads (Miltenyi Biotec) [4], [5]. This project was approved by the Australian Red Cross Blood Services and the University of New South Wales Human Ethics Committee. A written informed consent was obtained from each donor.

### Cell surface expression of LILRA2

Flow cytometric studies using freshly-isolated leukocytes were performed as described [5], [20]. In brief, purified leukocyte subsets were washed with cold PBS containing 0.05% NaN_3_ and 1% BSA (PAB buffer) and suspended in the same buffer at 2×10^6^/ml. Cells were then incubated for 30 min at room temperature with mAbs to LILRA2, or control mouse IgG_1_ (each at 5 µg/ml), washed twice in PAB buffer and incubated on ice for 45 min with 10 µl (10 µg/ml) FITC-conjugated F(ab′)_2_ goat anti-mouse IgG [F(ab′)_2_-specific] (Jackson ImmunoResearch Laboratories, West Grove, PA). Cells were fixed with 1% paraformaldehyde and analyzed by flow cytometry. In some experiments, LILRA2-expressing cells were characterized by co-staining with Alexa Fluor-488-conjugated anti-LILRA2 mAb (Zenon labeling technology, Molecular probes) and a combination of saturating amounts of flourochrome conjugated antibodies. In brief, anti-CD14-PE, anti-FcεRI-PE or combinations of CD56 Percp and CD19-APC or CD3-PE or CD4-Percp and CD8-APC were used to identify monocytes, basophils, NK cells, B cells and T cell subsets in PBMC respectively. Anti-CD9-PE and anti-CD16-PE were used to distinguish eosinophils and neutrophils in the PMN fraction of the gradient purified leukocytes. Isotype and flurochrome-matched negative control antibodies were added to cells stained with Alexa Fluor-488 conjugated IgG_1_ control mAb. Cells were fixed in 1% paraformaldehyde analysed using a four-colour FACS Calibur Flow cytometer (BD Biosciences).

### Activation of monocytes by LPS and/or LILRA2 cross-linking *in vitro*

Magnetic bead purified monocytes were activated for 18 h using 10 ng/ml of LPS (Sigma-Aldrich, St Louis, MO) or by cross-linking LILRA2 using anti-LILRA2 mAb as described [7], [21]. In brief, wells in a 96-well flat-bottom tissue culture plates (Costar® 3596, Corning, NY) were coated with 100 µL (50 µg/ml) of F(ab′)_2_ goat anti-mouse IgG, Fc-specific (Jackson ImmunoResearch Laboratories), in PBS overnight at 4°C. Following aspiration, 50 µl anti-LILRA2 mAb at 10 µg/ml in PBS+2.5% fraction V BSA (Boehringer, Mannheim, Germany) was added. Irrelevant mouse IgG_1_ or anti-MHC class I mAb were used as negative controls. Plates were incubated for 2 h at 37°C in 5% CO_2_ then rinsed with 0.9% NaCl before use. Monocytes (1×10^5^ cells) in 200 µl of RPMI 1640 supplemented with 10 mM HEPES (Sigma) and 0.1% BSA (Sigma) were added to each well and incubated for 18 h at 37°C and 5% CO_2_. Cell-free supernatants were collected for measurement of 18 cytokines using multiplex assay according to manufacturer's instruction (Bio-Plex™ Cytokine Assay, Bio-Rad Laboratories, Hercules, CA).

To assess the effects of simultaneous stimulation of monocytes with LPS and LILRA2, LILRA2 was cross-linked using anti-LILRA2 mAb in the presence or absence of 10 ng/ml of LPS for 18 h and cytokines measured by ELISA (R&D Systems, Minneapolis, MN, USA). Sequential activation of LILRA2 followed by LPS was performed to determine whether pre-activation of cells through LILRA2 affects responses to LPS. In brief, cells were cross-linked for 30 min with anti-LILRA2 coated plates followed by further treatment with or without 10 ng/ml of LPS for 18 h and cytokine levels in culture supernatants were then measured by ELISA (R&D Systems). Wells coated with irrelevant IgG_1_ or anti-MHC-I mAb with or without LPS were used as controls. Effects of LILRA2 cross-linking on cytokine production during shorter LPS treatment were assessed by pre cross-linking of cells with anti-LILRA2 for 30 min followed by a further 2 h or 6 h co-incubation. Selected cytokines in culture supernatants were then measured by ELISA.

### Modulation of TLR4 mRNA and protein expression in monocytes by LILRA2 cross-linking

Expression of TLR4 mRNA was analyzed at various time-points after LILRA2 cross-linking. In brief, 2×10^5^ monocytes were washed and re-suspended in culture media, RPMI 1640 supplemented with 10 mM HEPES (Sigma) and 5% heat inactivated autologous serum. Cells were stimulated by adding anti-LILRA2 or IgG_1_ control mAb (10 µg/ml), followed by cross-linking using goat anti-mouse Fcγ-specific F (ab)_2_ fragment secondary antibody (30 µg/ml, Jackson ImmunoResearch Laboratories) for 6, 12, 18 and 24 h at 37°C incubator with 5% CO_2_. Total RNA was extracted using Trizol (Invitrogen) from LILRA2 cross-linked monocytes and un-stimulated controls. Reverse transcription was performed on 1 µg RNA using SuperScript III First Strand Synthesis Supermix for qRT-PCR (Invitrogen) in a final volume of 20 µl. cDNA was then diluted to 50 µl in RNase and DNase free water. Aliquots (5 µl) of cDNA in a final volume of 25 µl were mixed with 1× SYBR GreenER qPCR SuperMix, 50 nM Rox dye and 200 nM of each TLR4 primer (forward 5′-ACTGCAGCTTCAACCGTATC-3′; reverse 5′-TAAAGGCTCTGCACACATCA-3′) [22] and 100 nM of β-actin primers (forward 5′-CATGTACGTTGCTATCCAGGC-3′; reverse 5′-CTCCTTAATGTCACGCACGAT-3′) [23]. Quantitative RT-PCR was performed on an ABI Prism 7700 Sequencer (Applied Biosystems, Foster City, CA). The amplification program was as follows: initialization at 50°C and 95°C for 2 minutes each followed by 40 cycles of denaturation at 95°C for 15 seconds and primer annealing/extension at 60°C for 60 seconds. The integrity of amplification was verified by a dissociation curve analysis and displayed a single melt peak for each product. The specificity of each reaction was further confirmed by detection of a single product of the expected size after agarose gel analysis. The same background subtractions and threshold values for all reactions were used to determine CT values. These standardized values were then used to obtain mean values of multiple experiments for statistical analysis. Results were expressed as relative ratios to the corresponding β-actin expression.

To assess the effects of LILRA2 cross-linking on TLR4 surface expression, cells were activated through LILRA2 for 6–48 h as described above and stained with directly FITC conjugated anti-TLR4 mAb and levels of expression determined by flow cytometry.

### Polystyrene-bead and bacterial phagocytosis by monocytes after LILRA2 cross-linking

The effect of LILRA2 stimulation on phagocytosis was determined using monocytes (2×10^5^) stimulated by LILRA2 cross-linking using anti-LILRA2 mAb for 12 h and 48 h followed by a single wash using RPMI 1640. To assess polystyrene-beads phagocytosis, 2 µm carboxylate polybeads (Polysciences, Warrington, PA) were first coated with purified human IgG (Sigma) using the BioMag® Maxi Carboxyl method according to the manufacturer's instructions (Polysciences). Beads (3×10^7^) were then biotinylated with Sulfo-NHS-LC-Biotin (0.3 mg/ml) as per manufacturer's instructions and stored at 4°C until use (Pierce, Rockford, IL). On the day of experiment, biotinylated beads were labeled with Streptavidin-conjugated Alexa 488 for 15 min, washed and re-suspended in RPMI 1640 supplemented with 10 mM HEPES (Sigma) and 0.1% BSA (Sigma). Beads were added onto LILRA2 cross-linked cells at a ratio of 10∶1 and incubated for 1 h at 37°C. Cells were washed twice in PAB, fixed in 1% paraformaldehyde in PBS then the percentage of monocytes that have ingested beads was determined by flow cytometry.

For bacteria phagocytosis, K12 *E. coli* (a gift from Prof. Hazel Mitchell, University of New South Wales, Australia) were labeled with pHrodo phagocytosis particle labeling kit according to the manufacturer's instruction (Molecular Probes, Eugene, OR). Labeled bacteria were lyophilized at 5 mg aliquots (∼2×10^9^ particles) and stored at −80°C until use. For subsequent experiments, an aliquot of the labeled *E. coli* was thoroughly re-suspended in 1 ml of Buffer B (Molecular Probes) and incubated on ice for 10 min. Half (0.5 ml) of the bacteria was pelleted by centrifugation at 500× g for 5 minutes, then opsonized with 0.5 ml of pooled human serum from 5 healthy donors for 30 min on ice [24] and the other half was left in Buffer B (non-opsonized). Opsonized and non-opsonized *E-coli* were added onto LILRA2 stimulated monocytes at a ratio of 20∶1 and incubated in a 37°C water bath for 15 min followed by 15 min incubation on ice. Cells were then sequentially washed with 1 ml cold Buffer B and 1 ml Buffer C (Molecular Probes), co-stained with FITC conjugated CD14 mAb and re-suspended in 300 µl of Buffer C. Percentages of monocytes that ingested bacteria were then determined using FACS Calibur flow cytometer by acquiring 10,000 events per tube. Pre-treatment of cells with 1 µM of latrunculin, an inhibitor of phagocytosis (Enzo Life Sciences, Farmingdale, NY) for 10 min in RPMI1640 media prior to the addition of micro beads or *E-coli* were used as controls.

## Results

### Surface expression of LILRA2 on peripheral blood leukocytes

**Figure 1:**
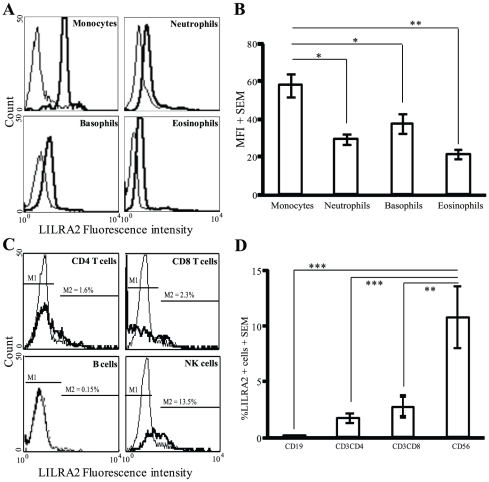
LILRA2 is differentially expressed on the surface of peripheral blood leukocytes. **A.** Flow cytometric analysis showing surface expression of LILRA2 on 100% of purified monocytes, neutrophils, eosinophils and basophils (bold lines, upper panel). In contrast, only small proportions of CD4+, CD8+ T cells or CD56+ NK cells surface expressed LILRA2 and no LILRA2 was expressed on CD19+ B cells (lower panel). **B.** The mean fluorescence intensity of LILRA2 staining (level of expression) was significantly higher in monocytes as compared to neutrophils, basophils or eosinophils. **C, D.** The proportion of LILRA2 expressing cells was significantly higher in NK cells as compared to T and B cells but there was no significant difference between CD4 and CD8 T cell subsets. Isotype and fluorochrome-matched negative control antibodies were used as negative controls (thin line). Error bars represent SEM of 15 independent experiments, *p<0.05, **p<0.01, ***p<0.001.

The expression pattern of LILRA2 on the surface of various leukocytes was determined by flow cytometry. LILRA2 is expressed on the surface of all peripheral blood monocytes, neutrophils, basophils and eosinophils with significantly higher levels expressed on monocytes (MFI, 58.3±6.1) and lowest on eosinophils (MFI, 21.7±2.4) (Figs. 1A, B) (n = 15). By contrast, B cells did not express LILRA2, and expression of LILRA2 on T cells was limited to less than 2.5% of the CD8^+^ or CD4^+^ cell subsets and approximately 10% of NK cells expressed low levels of LILRA2 (Figs. 1C, D). The level of LILRA2 expression was significantly higher in monocytes as compared to the other myeloid cells (Fig. 1B) and the proportion of LILRA2 positive NK cells was significantly higher when compared to T and B cells (Fig. 1D). These results may suggest that the primary role of LILRA2 may be regulation of leukocytes that are mainly involved in innate immune responses.

### Activation of monocytes by either LILRA2 cross-linking and/or LPS

**Figure 2:**
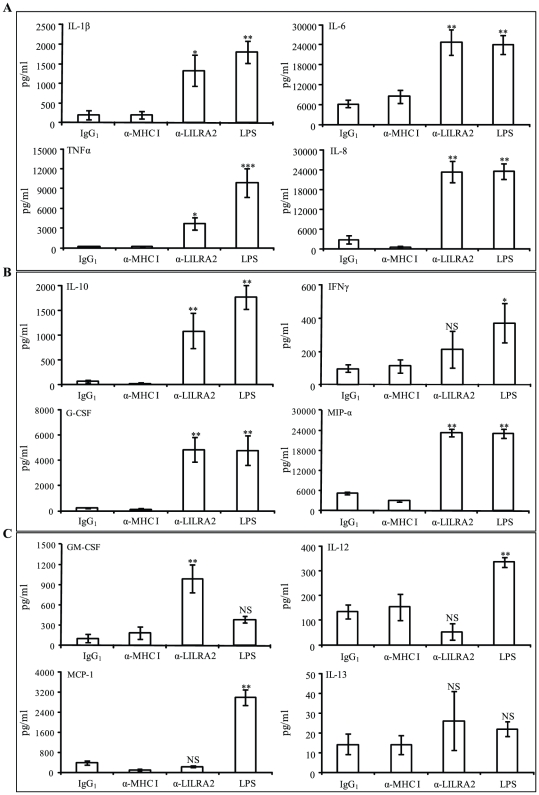
Activation of monocytes by LILRA2 cross-linking displays several differences and some similarity in cytokine production as compared to LPS. **A.** Activation of monocytes via LILRA2 cross-linking for 18 h significantly up-regulated the pro-inflammatory cytokines, IL-1β, IL-6, IL-8 and TNFα, similar to cells stimulated with LPS for 18 h, albeit lower levels of TNFα production after LILRA2 cross-linking. **B.** Monocytes activated through LILRA2 cross-linking also caused significant up-regulation of immune regulatory cytokines IL-10 and IFNγ, a T cell chemoattractant MIP-1α and a key cytokine in granulocyte development G-CSF at levels comparable to cells stimulated with LPS. **C.** In contrast, induction of GM-CSF and inhibition of IL-12 and no effects on MCP-1 after LILRA2 cross-linking was different to cells treated with LPS that showed significant increase in IL-12 and MCP-1 but minimal effects on GM-CSF. Neither LILRA2 cross-linking nor LPS treatment caused any significant modulation of IL-13 production. Cells treated with irrelevant IgG_1_ or anti-MHC class I mAb did not alter cytokine production. Cytokines in culture supernatants were assessed by Bioplex multi-cytokine assay in quadruplicates for each experiment. Error bars represent SEM of 4 independent experiments, *p<0.05, **p<0.01, ***p<0.001 anti-LILRA2 or LPS treated as compared to IgG_1_ treated control cells.

We first compared the cytokine profile of LILRA2 activation in monocytes as opposed to LPS stimulation using a multiplex cytokine assay. Activation of monocytes by LILRA2 cross-linking caused marked increase in GM-CSF production that was significantly higher (3-fold higher) compared to LPS stimulated cells but failed to induce IL-12 and MCP-1 production that were strongly up-regulated by LPS (Fig. 2). Interestingly, LILRA2 cross-linking induced similar amounts of IL-6, IL-8, G-CSF and MIP-1α but lower levels of TNFα (3596 vs 9871 pg/ml), IL-1β (1326 vs 1802 pg/ml), IL-10 (1080 vs 1754 pg/ml) and IFNγ (211 vs 370 pg/ml) compared to those stimulated with LPS (Fig. 2). Unlike LPS, LILRA2-mediated activation of monocytes only induced the production of IL-10, one of three immunomodulatory cytokines (IL-10, IL-12 and IFNγ) (Fig. 2). There was little or no effect on the expression of IL-13 (Fig. 2), IL-2, IL-4, IL-5, IL-17 and Eotaxin in cells treated with LPS or LILRA2 cross-linking (data not shown). These results suggest LILRA2 may have some overlapping and some distinct functions from TLR4.

**Figure 3:**
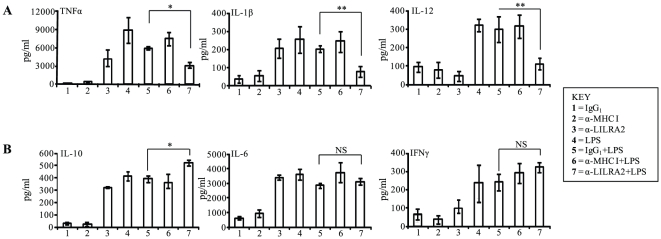
LPS-mediated cytokine production in monocytes is differentially modulated by concurrent activation by LILRA2 cross-linking. **A.** Simultaneous activation of monocytes with LPS and LILRA2 cross-linking for 18 h significantly inhibited TNFα, IL-1β and IL-12 production as compared to cells treated with LPS alone (4), LPS and IgG_1_ (5) or LPS and anti-MHC class I mAb (6). **B.** By contrast, this caused significant increase in IL-10 production and had little or no effect on IL-6 and IFNγ production. As expected cells treated with irrelevant IgG_1_ (1) or anti-MHC class I mAb (2) alone did not induce any cytokine production. Cytokine levels in culture supernatants were analysed by Bioplex multi-cytokine assay in quadruplicates for each experiment. Error bars represent SEM of 4 independent experiments, *p<0.05, **p<0.01 as compared to corresponding IgG_1_ and LPS treated controls.

**Figure 4:**
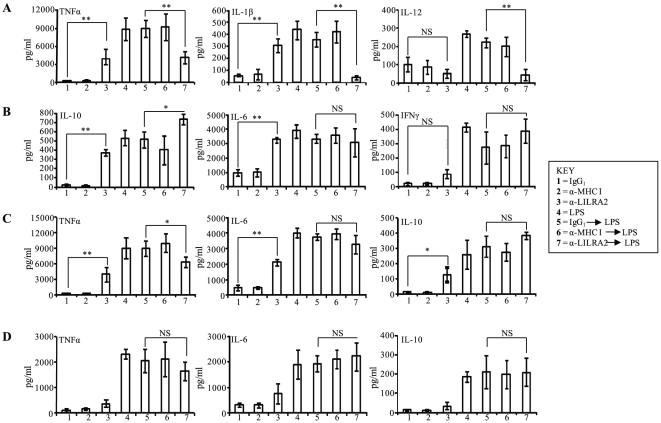
Activation of monocytes via LILRA2 cross-linking significantly modulates subsequent LPS-mediated production of selected cytokines. **A.** Monocytes pre-activated through LILRA2 cross-linking using anti-LILRA2 mAb for 30 min followed by 18 h stimulation with LPS produced significantly lower levels of TNFα, IL-1β and IL-12 as compared to cells treated with LPS alone (4), LPS and IgG_1_ (5) or LPS and anti-MHC class I mAb (6). **B.** Pre -activation of monocytes via LILRA2 had additive positive effect on subsequent LPS-mediated IL-10 production but did not affect IL-6 or IFNγ production. **C.** At 6 h time point, significant inhibition of TNFα production, modest increase in IL-10 and no change in IL-6 production was observed in response to LPS in cells cross-linked with anti-LILRA2. **D.** By contrast, the effects at 2 h time point were minimal for all three cytokines, although the inhibitory trend for TNFα was evident. Cytokine levels in culture supernatants were analysed by Bioplex multi-cytokine assay in quadruplicates for each experiment. Error bars represent SEM of 3 independent experiments, *p<0.05, **p<0.01, ***p<0.001.

To assess whether LILRA2 regulates TLR4 mediated production of selected cytokines in monocytes, LILRA2 on the surface of cells was cross-linked in the presence of 10 ng/ml of LPS. Co-stimulation of monocytes with LPS and LILRA2 cross-linking for 18 h produced significantly lower levels of TNFα (3171 vs 9871 pg/ml), IL-1β (75 vs 252 pg/ml) and IL-12 (112 vs 321 pg/ml) production (Fig. 3A) but had marginal additive effect on levels of IL-10 (531 vs 400 pg/ml) and IFNγ (323 vs 237 pg/ml) and no effect on IL-6 levels (3102 vs 3595 pg/ml) production (Fig. 3B) compared to anti-LILRA2 or LPS alone, indicating that LILRA2 may selectively inhibit TLR4 signaling. Cross-linking of cells with control mAbs alone showed no significant induction of any cytokines examined nor did they alter LPS-induced cytokine production (Fig. 3). Similarly, 30 min pre-stimulation of monocytes through LILRA2 cross-linking caused significant inhibition of subsequent LPS-induced TNFα, IL-12 and IL-1β production by two, four, and seven-fold respectively (Fig. 4A). There was no significant effect on subsequent LPS-induced IL-6 (3100 vs 3345 pg/ml), IL-10 (733 vs 514 pg/ml) or IFNγ (388 vs 273 pg/ml) production when compared to cells pre-treated with control IgG_1_ (Fig. 4B). To investigate if similar effects were observed at a shorter incubation with LPS, levels of 3 cytokines that showed different pattern of regulation at the 18 h time point were investigated (decrease, TNF; increase, IL-10; and no change, IL-6). LILRA2 cross-linking showed time dependent inhibition of subsequent LPS-induced TNFα production with an average of 19.6% inhibition at 2 h and 29.7% at 6 h (Fig. 4 C, D). This is lower than the 55% inhibition observed at 18 h time point (Fig. 4A). Interestingly, LILRA2 cross-linking followed by LPS treatment for 2 h had no effect but caused modest ∼23% increase in IL-10 production at 6 h as compared to the significant 42% increase observed at 18 h time point (Fig. 4). Minimal effect was observed on IL-6 production at both 2 h and 6 h time points (Fig. 4). Cross-linking with anti-LILRA2 alone for 2 h did not induce TNFα, IL-6 or IL-10 production, while there was significant induction of all 3 cytokines at 6 h and 18 h time points with maximum effects observed at 18 h (Fig. 4).

### Regulation of TLR4 mRNA and protein expression in monocytes after LILRA2 cross-linking

**Figure 5:**
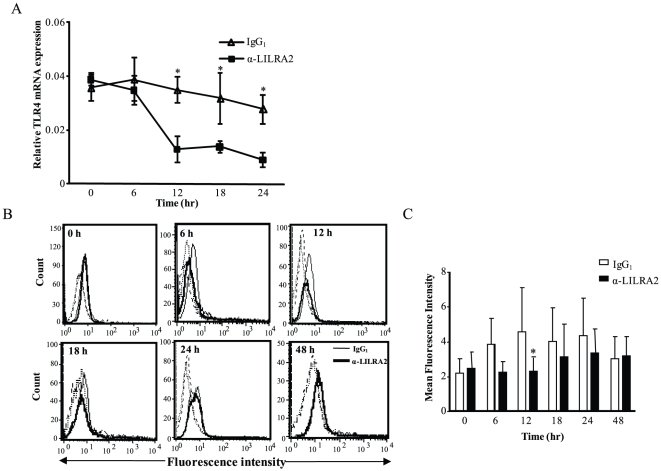
Cross-linking of LILRA2 on monocytes caused time dependent down-regulation of TLR4 mRNA and surface protein expression. **A.** Quantitative RT-PCR on mRNA extracted from purified monocytes that were activated through LILRA2 cross-linking caused down-regulation of TLR4 mRNA expression with significant changes observed at 12, 18 and 24 h but not at 6 h time point. **B.** Representative flow cytometry for the expression of TLR4 protein on the surface of monocytes activated via LILRA2 cross-linking for 6–48 hr. Dotted histograms on the left of each plot are cells stained with isotype matched negative control mAb. **C.** Summary of the mean fluorescence intensity (MFI) showing decreased expression of surface TLR4 on cells activated via LILRA2 cross-linking as compared to cells treated with IgG_1_ control with statistically significant difference seen at 12 h time point. Error bars represent SEM of 5 independent experiments, *p<0.05.

To determine whether the inhibitory effect of LILRA2 cross-linking was due to down-regulation of LPS-recognition receptors mainly TLR4, mRNA level of these receptor on monocytes was assessed. LILRA2 cross-linking consistently down-regulated TLR4 mRNA expression by up to 3fold at 12, 18 and 24 hour time points when compared to those treated with control IgG1 (Fig. 5A). Similarly, TLR4 protein expression on the surface of LILRA2 cross-linked cells was down-regulated at 6, 12 and 18 h time points with the highest and statistically significant effect observed 12 h after LILRA2 cross-linking (Fig. 5B, C). Interestingly, prolonged LILRA2 cross-linking (24–48 h) does not seem to show significant difference in TLR4 expression to control cells (Fig. 5C).

### Effects of LILRA2 cross-linking on phagocytosis of IgG coated micro-beads and serum opsonised and non-opsonised *E coli*

**Figure 6:**
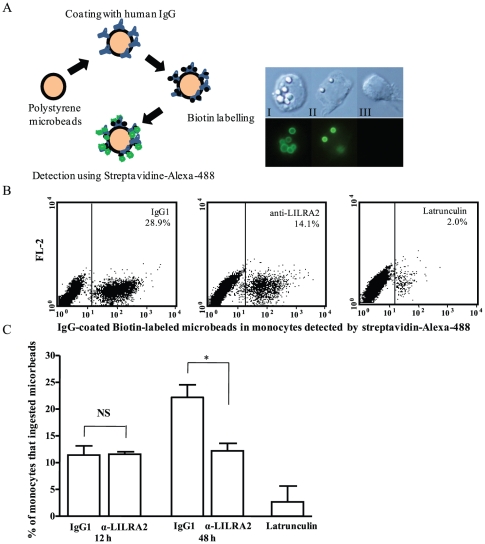
Prolonged cross-linking of LILRA2 on monocytes significantly reduced phagocytosis of human IgG-coated polystyrene beads. **A.** Schematic representation of human IgG-coated biotin-labeled polystyrene microbeads detected by streptavidine-Alexa-488 green fluorescence (left) and representative phase contrast and fluorescence images of monocytes on the right showing uptake of variable numbers of beads by monocytes (I, II) or none (III) after 1 h incubation with the biotin-labeled-IgG-coated beads. **B.** Representative Flow cytometry showing decreased phagocytosis of human IgG-coated-biotin-labeled beads by monocytes that were activated via LILRA2 cross-linking for 48 h and incubated with the beads for 1 hr as compared to cells treated with control IgG_1_. Treatment with Latrunculin, a pharmacological inhibitor of phagocytosis but not surface attachment was used as a relevant control (right). **C.** Summary flow cytometric analysis of 1 hr bead phagocytosis showing significant decrease of uptake by monocytes that were activated via LILRA2 cross-linking for 48 h but not 12 h as compared to control IgG_1_ treated cells. Data from the Latrunculin treated cells indicate that less than 2% of bead positive cells might be due to non-specific attachment of beads on the cell surface. Error bars represent SEM of 4 independent experiments, *p<0.05.

**Figure 7:**
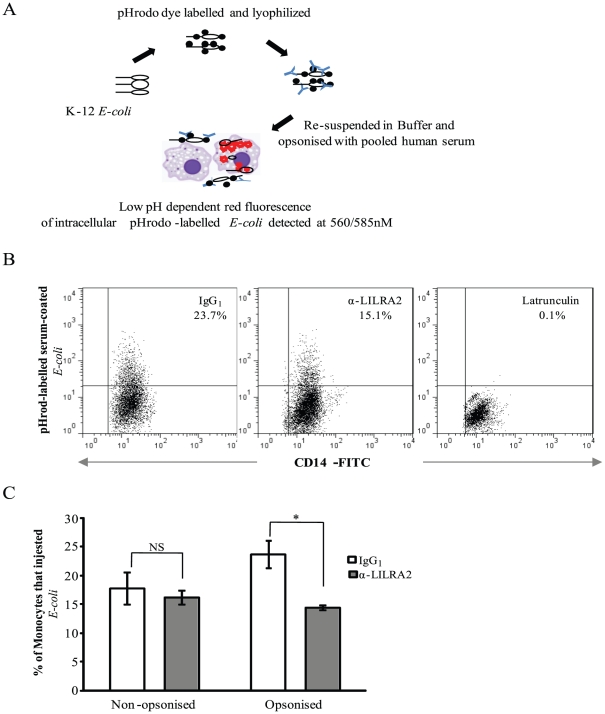
Prolonged cross-linking of LILRA2 on monocytes significantly reduced phagocytosis of serum opsonized but not non-opsonized *E-coli*. **A.** Schematic representation of serum opsonized pHrodo dye-labeled *E-coli* that fluoresce at low pH within phagocytic cells and detected by Flow cytometry. **B.** Representative Flow cytometry showing phagocytosis of serum opsonized *E-coli* by CD14+ monocytes that were activated via LILRA2 cross-linking, control IgG_1_ for 48 h or pretreated with Latrunculin for 10 min. **C.** Summary flow cytometric analysis of 15 min *E-coli* phagocytosis showing significant decrease of uptake of opsonized but not non-opsonized bacteria by monocytes that were activated via LILRA2 cross-linking for 48 h as compared to control IgG_1_ treated cells. Error bars represent SEM of 3 independent experiments, *p<0.05.

To assess whether LILRA2 modulates phagocytosis of IgG-coated micro-beads or *E-coli*, monocytes were activated via LILRA2 cross-linking for 12 h and 48 h. Activation of monocytes via LILRA2 cross-linking for 12 h had no effect on phagocytosis of IgG coated micro-beads (Fig. 6A, C). However, activation of these cells through LILRA2 cross-linking for 48 h significantly reduced bead phagocytosis by 30–50% as compared to cells treated with control mAb (Fig. 6B, C). Similarly, activation of monocytes through LILRA2 cross-linking for 48 h (Fig. 7) but not 12 h (data not shown) has significantly inhibited phagocytosis of serum opsonised *E coli* by 35–40% while having little effect (5–10% inhibition) in uptake of non-opsonised *E coli*, despite the latter being phagocytised by up to 17% of monocytes (Fig. 7). These results indicate selective and delayed effects of LILRA2 on Fc receptor dependent bacterial and particle phagocytosis. Treatment of cells with a known inhibitor of phagocytosis (latrunculin) showed minimal bead or bacterial phagocytosis (Figs. 6, 7).

## Discussion

There is ample *in vitro* evidence [4]–[6] and clinical correlation studies [8], [9], [25]–[27] that indicate LILRA2 as a key immune regulatory molecule. For example, LILRA2 is abundantly expressed in diseases characterized by chronic inflammation [7]–[9], [25]–[27] and *in vitro* cross-linking of this receptor on monocytes, macrophages [6], eosinophils [4] and basophils [5] induces production of several mediators involved in immune regulation. Moreover, LILRA2 has been shown to inhibit dendritic cells differentiation and reduce antigen presentation to T cells *in vitro* [6]. The predominant expression LILRA2 in mono-myeloid and NK cells shown in this study may indicate that the primary role of this receptor may be in the regulation of innate immune responses. Interestingly, a high level of LILRA2 is constitutively co-expressed on monocytes with other key innate immune receptors to LPS, TLR4 and CD14 and receptors involved in phagocytosis of pathogens such as FcγRI [14] (Fig. 1). However, whether activation of monocytes through LILRA2 generates responses different to LPS and/or whether LILRA2 modulates TLR4 and FcγRI-mediated monocyte activation remains unknown.

Here we show that LILRA2 cross-linking on the surface of monocytes promotes strong induction of several pro-inflammatory cytokines to levels comparable with those induced by LPS, indicating overlapping effects. However, LILRA2 cross-linking, unlike LPS activation, did not induce IL-12 or MCP-1 production and caused production of significantly higher levels of GM-CSF than LPS (Fig. 2). These results illustrate major differences in LPS versus LILRA2-mediated activation of monocytes. Moreover, an increase in the ratio of IL-10 to IL-12 (20∶1) in culture supernatants of monocytes activated through LILRA2 as compared to those treated with LPS (5∶1) strongly indicate responses to LILRA2 may be biased towards Th2 type (Fig. 2) [9], [28]. Furthermore, our findings showed that LILRA2 cross-linking significantly reduced delayed Fc receptor dependent phagocytosis of micro beads and *E-coli* (Figs. 6, 7) but did not affect early uptake of particles or bacteria. These findings support the notion that LILRA2-mediated activation of macrophages may inhibit uptake of pathogens in chronic inflammation. These findings are in agreement with studies showing that increased LILRA2 expression on macrophages was strongly associated with lepromatous leprosy, a disease characterized by increased IL-10 to IL-12 ratios and Th2-mediated inflammatory responses together with poor bacterial clearance [9]. In contrast, patients with tuberculoid leprosy with minimal inflammation expressed low levels of LILRA2 and demonstrated strong Th-1 responses that were effective in mycobacterial killing [9]. Consistent with Th2 biased responses, LILRA2 cross-linking on the surface of alveolar macrophages was shown to dramatically reduce bactericidal activity to *Mycobacterium tuberculosis* [6]. Moreover, excessive production of GM-CSF in response to LILRA2 cross-linking may drive differentiation of monocytes into immature dendritic cells that poorly present antigen [29], [30].

Increased production of GM-CSF, but not MCP-1, in response to LILRA2 cross-linking may also represent a new mechanism that encourages *in situ* differentiation of monocytes but avert recruitment of blood-borne monocytes, thus preventing excessive inflammation [31], [32]. Potential inhibition of *de novo* recruitment of monocytes by LILRA2 together with its ability to inhibit LPS-induced pro-inflammatory cytokine production but up-regulate suppressive cytokines strongly suggests LILRA2 is a key regulator of the innate immune responses. Consistent with this proposal, we showed that cross-linking of LILRA2 on the surface of monocytes prior to LPS stimulation or simultaneous activation of cells through LILRA2 and TLR4 caused significant inhibition of pro-inflammatory cytokines while marginally enhancing the production of immune regulatory cytokines (Fig. 3, 4).

We show that LILRA2 cross-linking on monocytes prior to LPS stimulation, or simultaneous activation through LILRA2 and TLR4, significantly reduced production of pro-inflammatory cytokines and IL-12 (Fig. 3, 4). By contrast, LILRA2 cross-linking had positive additive effect on the production of the anti-inflammatory cytokine, IL-10 (Fig. 3, 4), the most significant effects observed at later time points (Fig. 4). Although inhibition of cellular activation by ITIM-containing inhibitory receptors is well established [33], [34], recent studies show that ITAM-containing activating receptors including a member of LILR family (LILRA4) [35], [36], may also propagate inhibitory signals against unrelated activating receptors [37]–[40]. The ITAM containing signaling motifs of these receptors are designated as inhibitory ITAM or ITAMi [39], [41]. ITAMi exert inhibitory effects only when stimulated in concert with unrelated activating receptor [40]. This is consistent to our results that showed that LILRA2 exerts its inhibitory effects only in the presence of LPS that engage TLR4 (Figs. 3, 4) or IgG-coated beads and serum opsonized *E-coli* that engage FcγRI (Figs. 6, 7). However, the underlying mechanisms for these inhibitory effects by ITAM-containing receptors such as LILRA2 are not fully elucidated. Here we show that cross-linking of LILRA2 on monocytes caused down-regulation of TLR4 mRNA and protein (Fig. 6), which may in part but not exclusively explain the selective reduced LPS responses seen with these cells (Figs. 3, 4). Induction of immunosuppressive mediators such as IL-10 (Fig. 2) in response to LILRA2 cross-linking may also contribute to the suppression of TLR4-mediated monocyte functions. It is noteworthy that cross-linking with anti-LILRA2 alone for 2 h had little effect in the production of IL-10, while there was significant induction at 6 h and 18 h time points (Fig. 4). This suggests that LILRA2-induced IL-10 might contribute to the delayed suppressive effects on LPS-mediated pro-inflammatory cytokine production. However detailed immunological and biochemical mechanisms for the selective LILRA2-mediated inhibitory effects on TLR signaling requires further investigation. These may include effects of LILRA2-cross-linking on specific pathways that regulate production of the affected cytokines (Fig. 3, 4) [41]–[44] and investigation on whether LILRA2 induces other inhibitory molecules that are known to strongly suppress TLR2 and TLR4 signaling including STAT3, SOCS3, A20 and ABIN-3 [36]. Furthermore, whether LILRA2 activation recruits inhibitory phosphatases such as SHP-1 that can abrogate LPS-responses [45] remains to be investigated.

Taken together we demonstrate that LILRA2 is a potent and highly selective activating receptor as well as preferential inhibitor of TLR4-mediated pro-inflammatory cytokine production and FcγRI-dependent phagocytosis. Therefore, effective activation of monocytes via LILRA2 may tightly regulate their responses to intracellular and extracellular pathogens and determine the nature and magnitude of the innate immune responses. Despite these overwhelming evidences for its key immune regulatory functions, the significance of LILRA2 *in vivo* is not well established, primarily due to lack of knowledge of its natural ligands. Thus, identification of ligands and characterizing their interaction with LILRA2 would further strengthen our understanding on the role of this molecule *in vivo*.
