# Correction: LILRA2 Selectively Modulates LPS-Mediated Cytokine Production and Inhibits Phagocytosis by Monocytes

**DOI:** 10.1371/annotation/f4475cbd-3518-46cd-bb6b-a326008af388

**Published:** 2012-08-07

**Authors:** Hao K. Lu, Ainslie Mitchell, Yasumi Endoh, Taline Hampartzoumian, Owen Huynh, Luis Borges, Carolyn Geczy, Katherine Bryant, Nicodemus Tedla

There was an error in affiliation 1 for authors Hao K. Lu, Ainslie Mitchell, Yasumi Endoh, Taline Hampartzoumian, Owen Huynh,Carolyn Geczy, Katherine Bryant, and Nicodemus Tedla. Affiliation 1 should be: Inflammation and Infection Research Centre, School of Medical Sciences, Department of Pathology, University of New South Wales, Australia 

